# Right Iliac Artery—Inferior Vena Cava Arteriovenous Fistula

**DOI:** 10.1055/s-0038-1639611

**Published:** 2018-07-27

**Authors:** Umberto G. Rossi, Pierluca Torcia, Gian Andrea Rollandi, Maurizio Cariati

**Affiliations:** 1 Department of Diagnostic Imaging - Interventional Radiology Unit, Galliera Hospital, Genova, Italy; 2 Department of Diagnostic Science - Radiology and Interventional Radiology Unit, ASST Santi Paolo and Carlo Hospital, Milano, Italy; 3 Department of Diagnostic Imaging - Radiology Unit, Galliera Hospital, Genova, Italy

**Keywords:** right iliac artery, inferior vena cava, arteriovenous fistula, pseudoaneurysm, elderly patient (aging)

## Abstract

This report describes a case of elderly patient with right iliac artery fissured pseudoaneurysm, leading to inferior vena cava arteriovenous fistula that was treated by covered stent device deployed at the level of the right iliac pathologic segment.

A 77-year-old male patient underwent open repair aorto-bi-iliac graft for abdominal aortic aneurysm (AAA) 7 years ago. On clinical follow-up, an abdominal bruit was observed. The patient underwent contrast medium-enhanced multidetector computed tomography (MD-CT) that revealed in the arterial phase, at the level of the right iliac anastomosis, the presence of fissured pseudoaneurysm (arrowhead in Fig. 1 ) in communication with the proximal third of the inferior vena cava, with a rapid contrast filling of an enlarged inferior vena cava (shown by * in Fig. 1 ). So, a diagnosis of right iliac artery–inferior vena cava arteriovenous fistula (AVF) was made. After multidisciplinary agreement, the patient underwent digital subtraction angiography that confirmed the diagnosis of fissured pseudoaneurysm (arrowhead in Fig. 2 ), leading to right iliac artery–inferior vena cava AVF with inferior vena cava enlargement (shown by * in Fig. 2 ). Consequently, a covered stent device was deployed as arterial coverage at the level of the fissured pseudoaneurysm of the right iliac anastomosis with the resolution of the AVF. The patient at 1-year follow-up demonstrated clinical and technical successes.

**Fig. 1:**
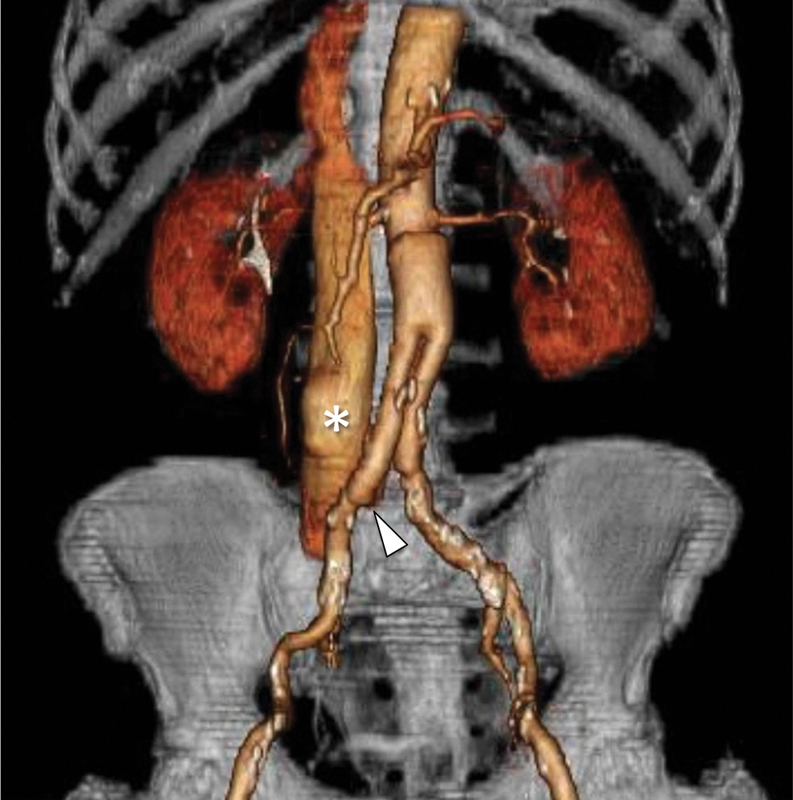
Abdominal contrast medium-enhanced multidetector computed tomography coronal volume rendering technique reconstruction that shows, at the level of the right iliac anastomosis, the presence of fissured pseudoaneurysm (arrowhead) in communication with the proximal third of the inferior vena cava (*).

**Fig. 2:**
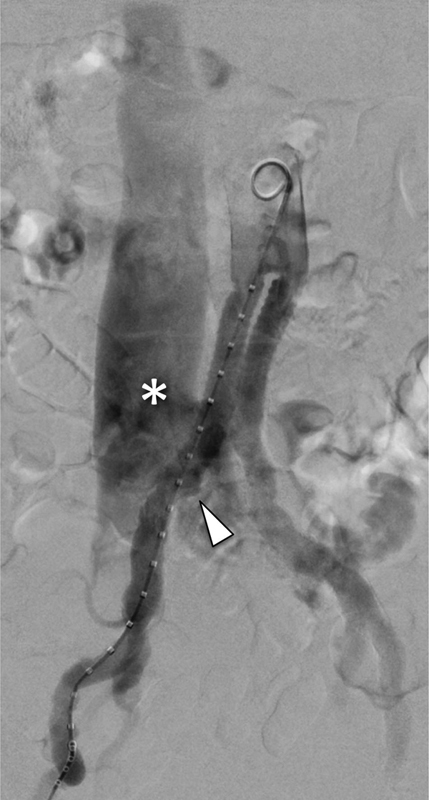
Abdominal digital subtraction angiography that confirms the fissured pseudoaneurysm (arrowhead), leading to right iliac artery—inferior vena cava (*) arteriovenous fistula.

Iliac artery–inferior vena cava or iliac vein AVF, due to iliac anastomotic pseudoaneurysm, is a rare clinical complication after AAA open repair. 1 Contrast medium-enhanced MD-CT is the first-line imaging modality to confirm the diagnosis and to plan the possible repair procedure in a suspected iliac artery–inferior vena cava or iliac vein AVF. 2 AVF as complication of anastomotic pseudoaneurysm must be treated to prevent its possible rupture and cardiac decompensation due to left-to-right shunting. Treatment options include endovascular techniques or open surgical management. Nowadays, endovascular techniques are the preferred modality for most clinicians. 3

## References

[1] Fukushima R B Wolosker N Benitti D A Puech-Leão P Endovascular treatment for iliac artery pseudoaneurysm with arteriovenous fistula after abdominal aortic aneurysm open repair Clinics (Sao Paulo) 2011 66 08 1499 1500 21915508 10.1590/S1807-59322011000800033 PMC3161236

[2] Fuentes-Orrego J M Pinho D Kulkarni N M Agrawal M Ghoshhajra B B Sahani D V New and evolving concepts in CT for abdominal vascular imaging Radiographics 2014 34 05 1363 1384 25208285 10.1148/rg.345130070

[3] Nakad G AbiChedid G Osman R Endovascular treatment of major abdominal arteriovenous fistulas: a systematic review Vasc Endovascular Surg 2014 48(5-6):388 395 24973241 10.1177/1538574414540485

